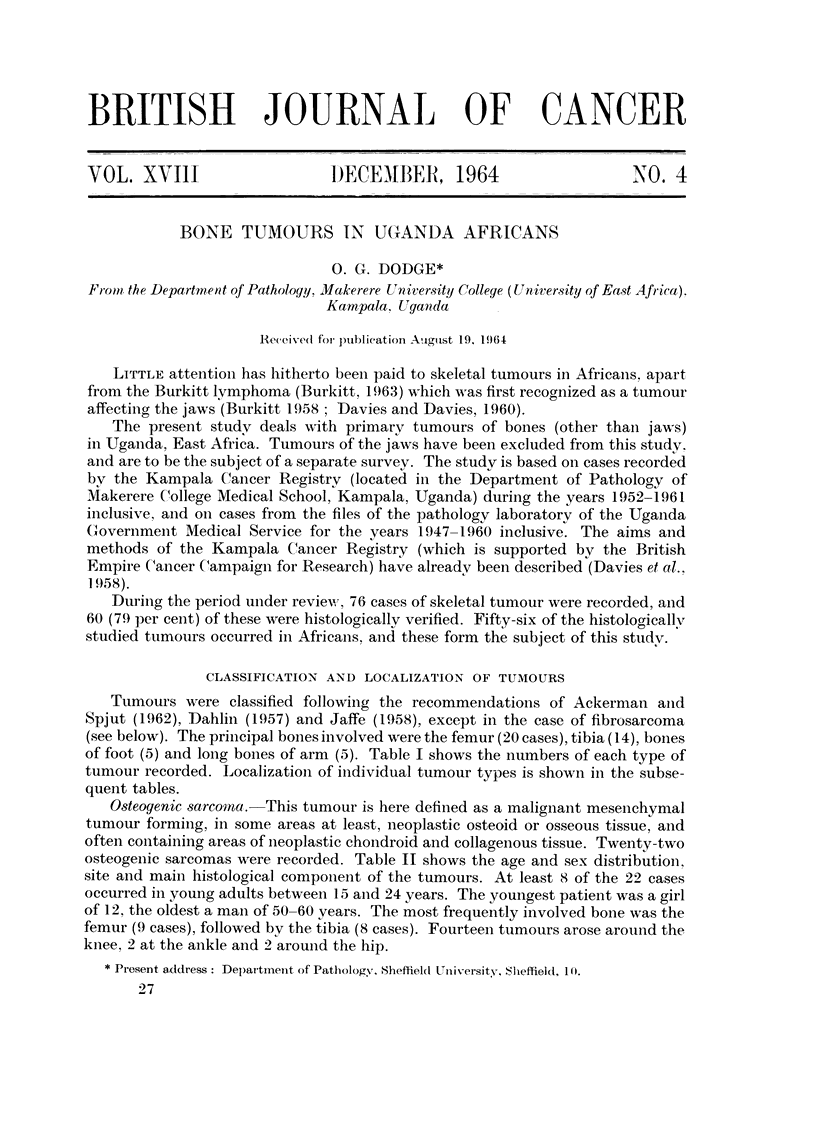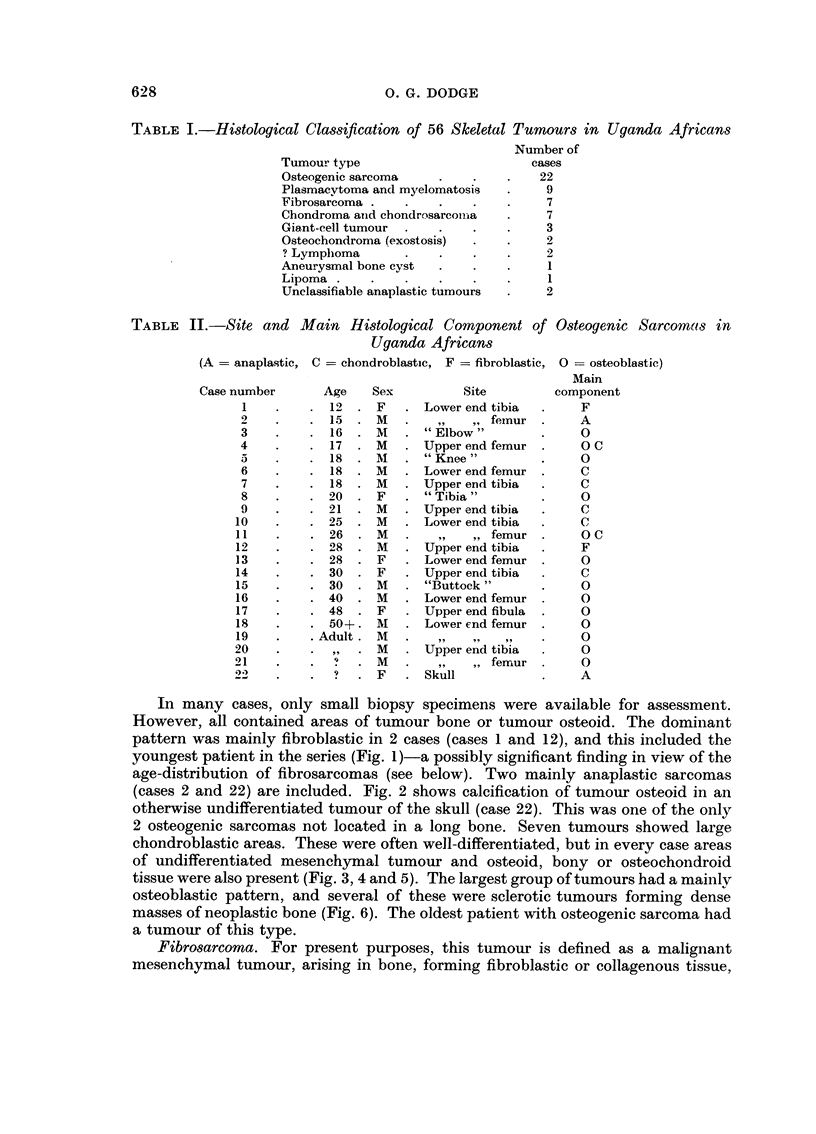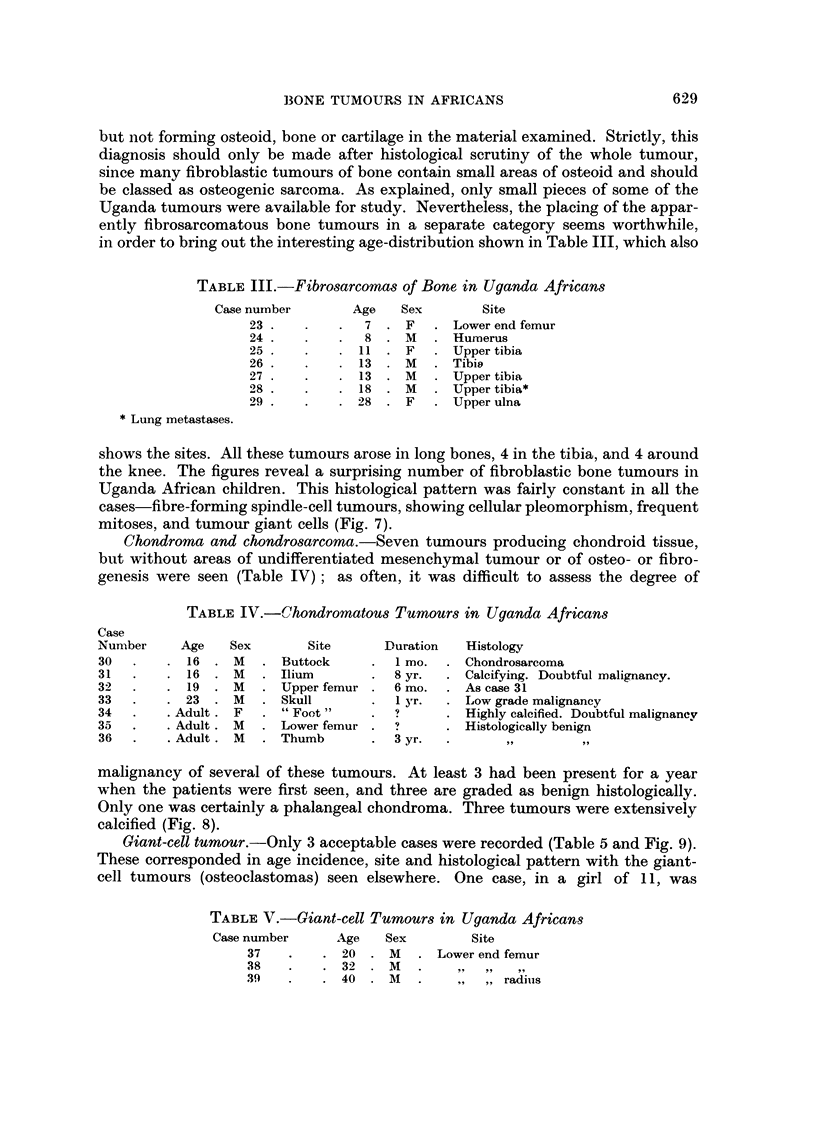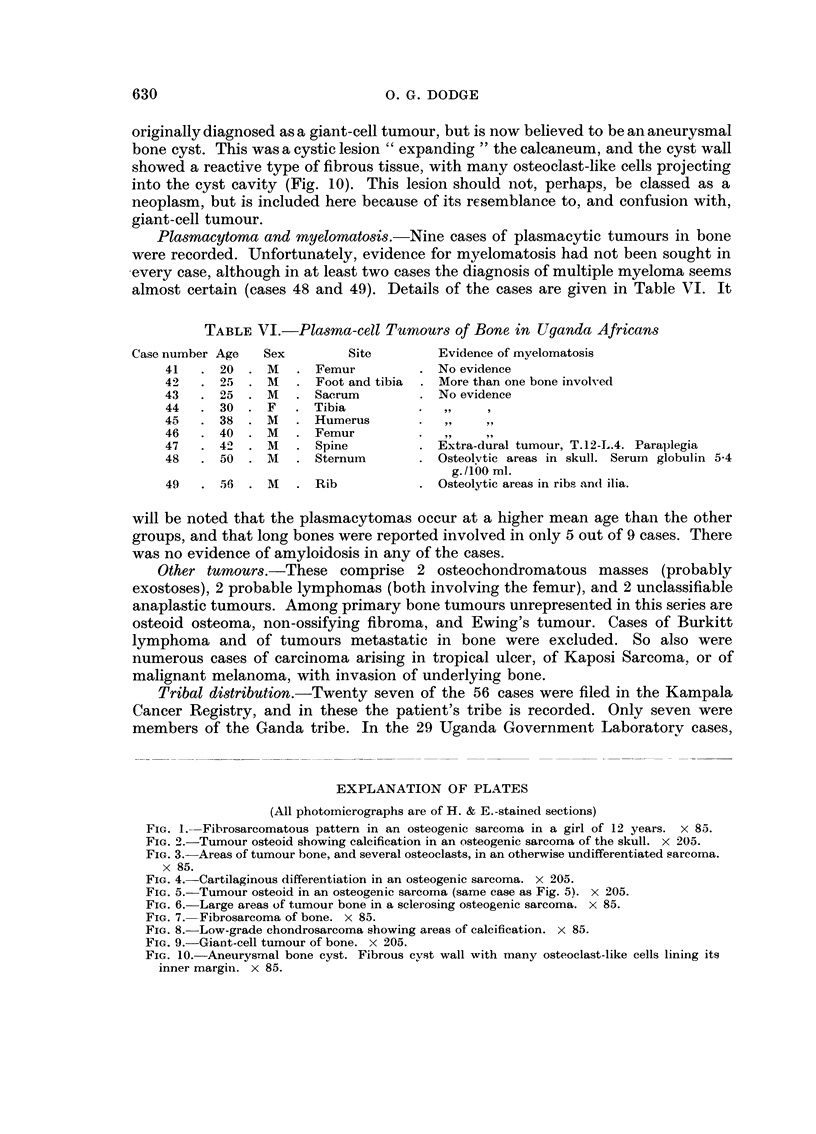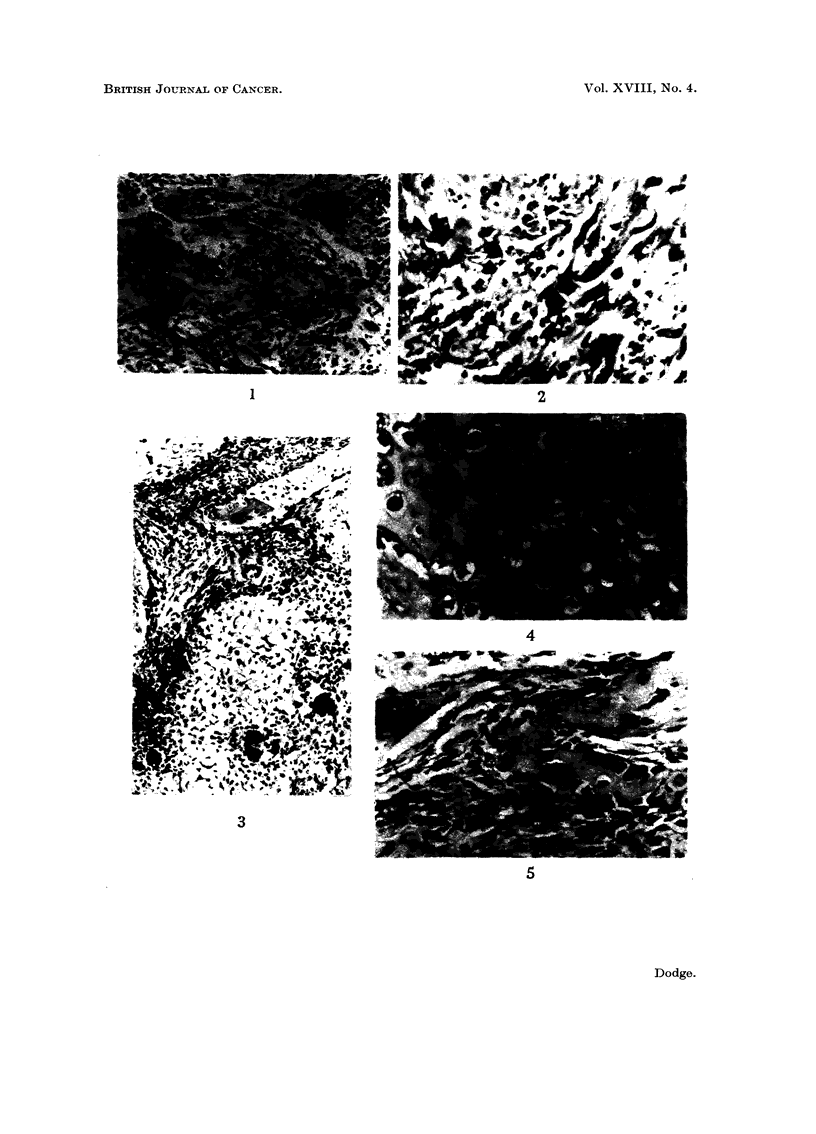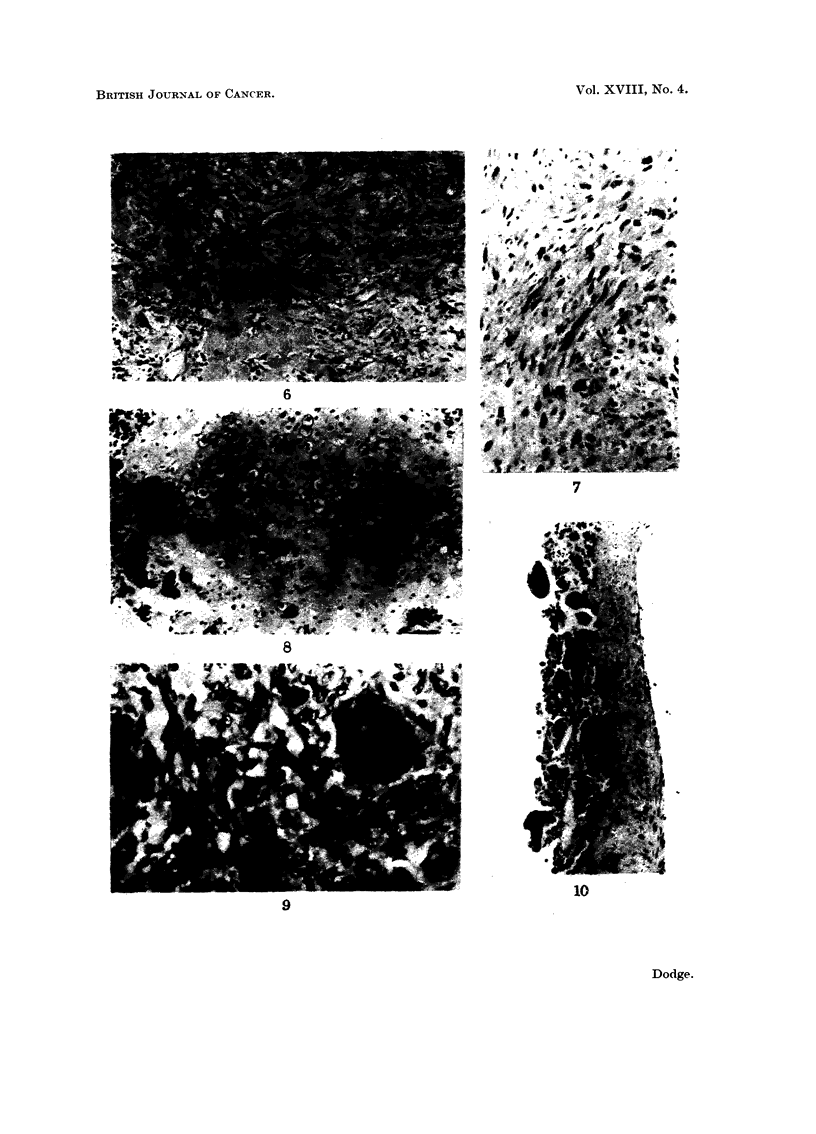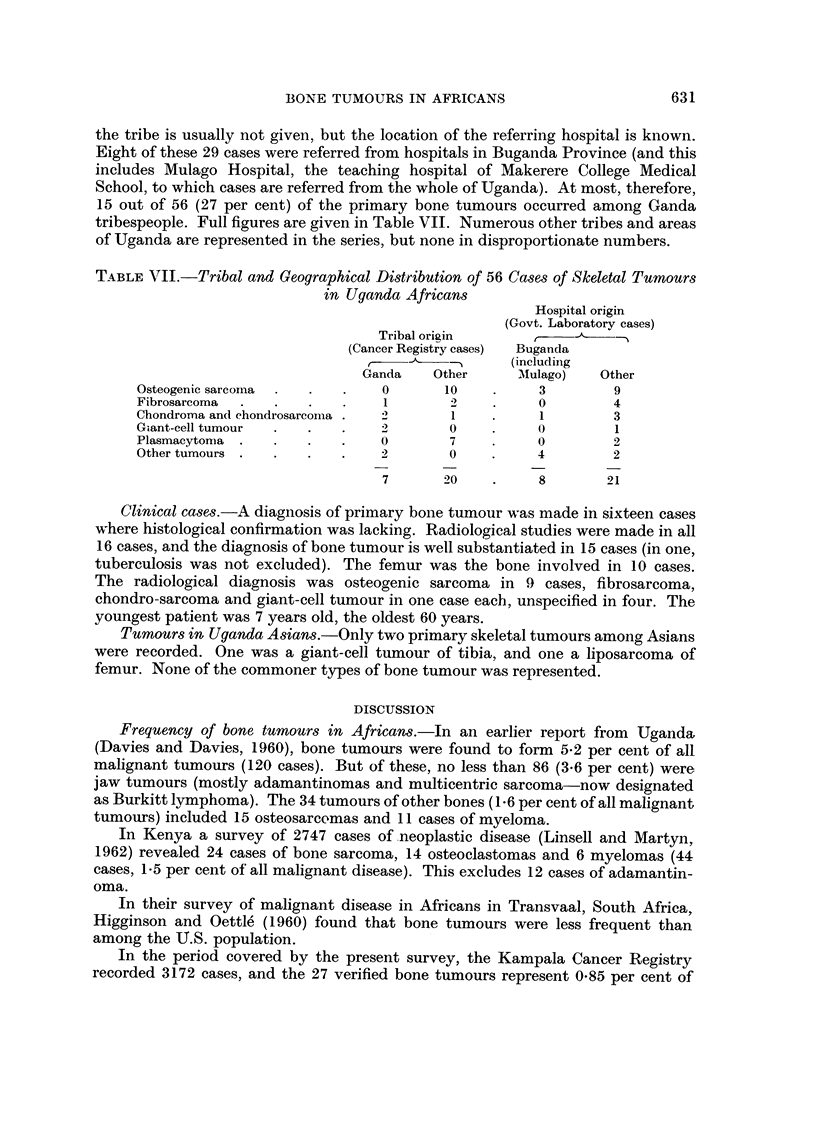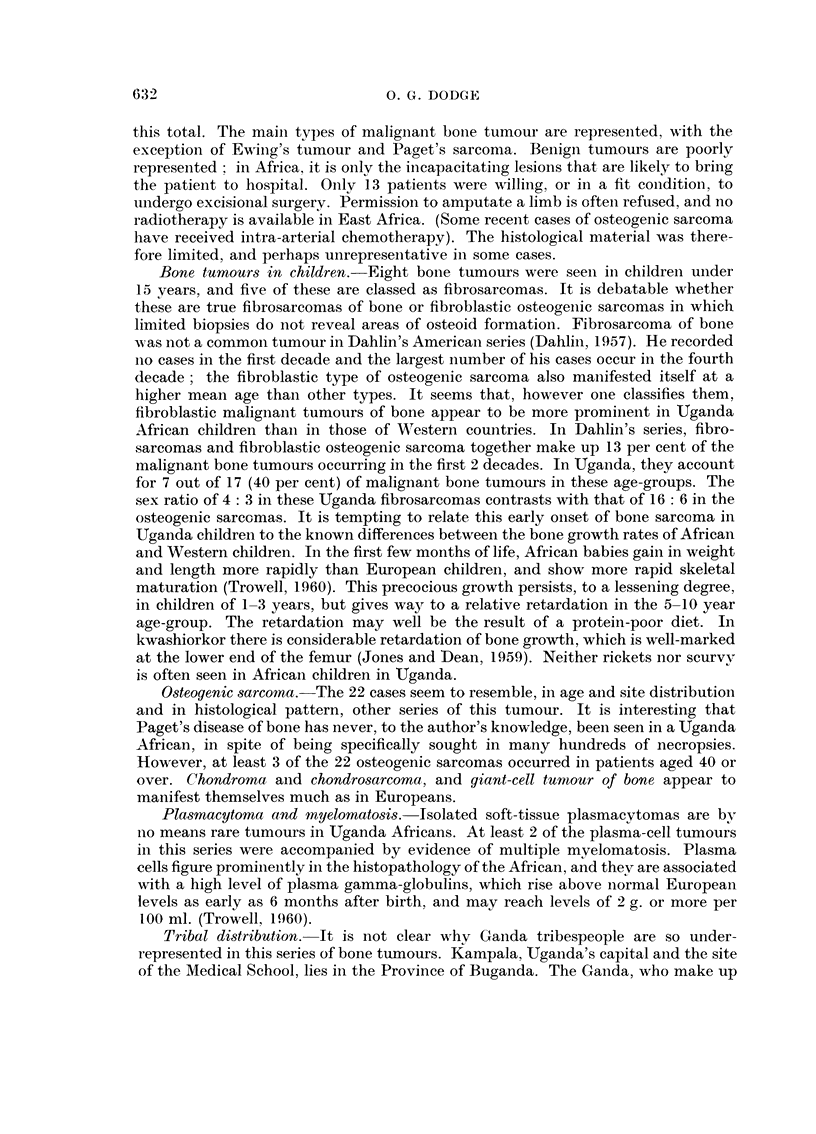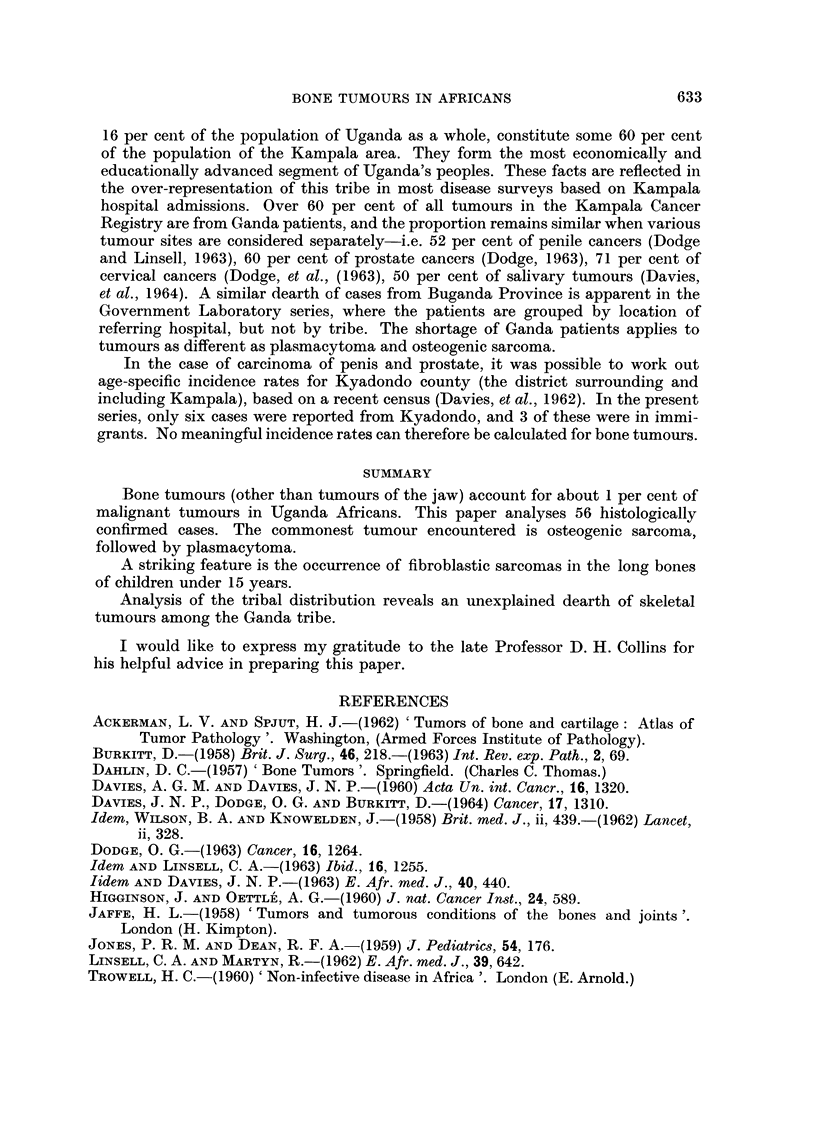# Bone Tumours in Uganda Africans

**DOI:** 10.1038/bjc.1964.72

**Published:** 1964-12

**Authors:** O. G. Dodge

## Abstract

**Images:**


BRITISH JOURNAL OF CANCER

VOL. XVIII DECEMBER, 1964 NO. 4

### BONE TUMOURS IN UGANDA AFRICANS

O. G. DODGE*

*From the Department of Pathology, Makerere University College (University of East Africa), Kampala, Uganda*

Received for publication August 19, 1964

LITTLE attention has hitherto been paid to skeletal tumours in Africans, apart from the Burkitt lymphoma (Burkitt, 1963) which was first recognized as a tumour affecting the jaws (Burkitt 1958 ; Davies and Davies, 1960).

The present study deals with primary tumours of bones (other than jaws) in Uganda, East Africa. Tumours of the jaws have been excluded from this study, and are to be the subject of a separate survey. The study is based on cases recorded by the Kampala Cancer Registry (located in the Department of Pathology of Makerere College Medical School, Kampala, Uganda) during the years 1952–1961 inclusive, and on cases from the files of the pathology laboratory of the Uganda Government Medical Service for the years 1947–1960 inclusive. The aims and methods of the Kampala Cancer Registry (which is supported by the British Empire Cancer Campaign for Research) have already been described (Davies *et al.*, 1958).

During the period under review, 76 cases of skeletal tumour were recorded, and 60 (79 per cent) of these were histologically verified. Fifty-six of the histologically studied tumours occurred in Africans, and these form the subject of this study.

### CLASSIFICATION AND LOCALIZATION OF TUMOURS

Tumours were classified following the recommendations of Ackerman and Spjut (1962), Dahlin (1957) and Jaffe (1958), except in the case of fibrosarcoma (see below). The principal bones involved were the femur (20 cases), tibia (14), bones of foot (5) and long bones of arm (5). Table I shows the numbers of each type of tumour recorded. Localization of individual tumour types is shown in the subsequent tables.

*Osteogenic sarcoma.*—This tumour is here defined as a malignant mesenchymal tumour forming, in some areas at least, neoplastic osteoid or osseous tissue, and often containing areas of neoplastic chondroid and collagenous tissue. Twenty-two osteogenic sarcomas were recorded. Table II shows the age and sex distribution, site and main histological component of the tumours. At least 8 of the 22 cases occurred in young adults between 15 and 24 years. The youngest patient was a girl of 12, the oldest a man of 50–60 years. The most frequently involved bone was the femur (9 cases), followed by the tibia (8 cases). Fourteen tumours arose around the knee, 2 at the ankle and 2 around the hip.

* Present address : Department of Pathology, Sheffield University, Sheffield, 10.

TABLE I.—*Histological Classification of 56 Skeletal Tumours in Uganda Africans*

| Tumour type | Number of cases |
|---|---|
| Osteogenic sarcoma | 22 |
| Plasmacytoma and myelomatosis | 9 |
| Fibrosarcoma | 7 |
| Chondroma and chondrosarcoma | 7 |
| Giant-cell tumour | 3 |
| Osteochondroma (exostosis) | 2 |
| ? Lymphoma | 2 |
| Aneurysmal bone cyst | 1 |
| Lipoma | 1 |
| Unclassifiable anaplastic tumours | 2 |

TABLE II.—*Site and Main Histological Component of Osteogenic Sarcomas in Uganda Africans*

(A = anaplastic, C = chondroblastic, F = fibroblastic, O = osteoblastic)

| Case number | Age | Sex | Site | Main component |
|---|---|---|---|---|
| 1 | 12 | F | Lower end tibia | F |
| 2 | 15 | M | ,, ,, femur | A |
| 3 | 16 | M | " Elbow " | O |
| 4 | 17 | M | Upper end femur | O C |
| 5 | 18 | M | " Knee " | O |
| 6 | 18 | M | Lower end femur | C |
| 7 | 18 | M | Upper end tibia | C |
| 8 | 20 | F | " Tibia " | O |
| 9 | 21 | M | Upper end tibia | C |
| 10 | 25 | M | Lower end tibia | C |
| 11 | 26 | M | ,, ,, femur | O C |
| 12 | 28 | M | Upper end tibia | F |
| 13 | 28 | F | Lower end femur | O |
| 14 | 30 | F | Upper end tibia | C |
| 15 | 30 | M | "Buttock " | O |
| 16 | 40 | M | Lower end femur | O |
| 17 | 48 | F | Upper end fibula | O |
| 18 | 50+ | M | Lower end femur | O |
| 19 | Adult | M | ,, ,, ,, | O |
| 20 | ,, | M | Upper end tibia | O |
| 21 | ? | M | ,, ,, femur | O |
| 22 | ? | F | Skull | A |

In many cases, only small biopsy specimens were available for assessment. However, all contained areas of tumour bone or tumour osteoid. The dominant pattern was mainly fibroblastic in 2 cases (cases 1 and 12), and this included the youngest patient in the series (Fig. 1)—a possibly significant finding in view of the age-distribution of fibrosarcomas (see below). Two mainly anaplastic sarcomas (cases 2 and 22) are included. Fig. 2 shows calcification of tumour osteoid in an otherwise undifferentiated tumour of the skull (case 22). This was one of the only 2 osteogenic sarcomas not located in a long bone. Seven tumours showed large chondroblastic areas. These were often well-differentiated, but in every case areas of undifferentiated mesenchymal tumour and osteoid, bony or osteochondroid tissue were also present (Fig. 3, 4 and 5). The largest group of tumours had a mainly osteoblastic pattern, and several of these were sclerotic tumours forming dense masses of neoplastic bone (Fig. 6). The oldest patient with osteogenic sarcoma had a tumour of this type.

*Fibrosarcoma.* For present purposes, this tumour is defined as a malignant mesenchymal tumour, arising in bone, forming fibroblastic or collagenous tissue, but not forming osteoid, bone or cartilage in the material examined. Strictly, this diagnosis should only be made after histological scrutiny of the whole tumour, since many fibroblastic tumours of bone contain small areas of osteoid and should be classed as osteogenic sarcoma. As explained, only small pieces of some of the Uganda tumours were available for study. Nevertheless, the placing of the apparently fibrosarcomatous bone tumours in a separate category seems worthwhile, in order to bring out the interesting age-distribution shown in Table III, which also

TABLE III.—*Fibrosarcomas of Bone in Uganda Africans*

| Case number | Age | Sex | Site |
|---|---|---|---|
| 23 | 7 | F | Lower end femur |
| 24 | 8 | M | Humerus |
| 25 | 11 | F | Upper tibia |
| 26 | 13 | M | Tibia |
| 27 | 13 | M | Upper tibia |
| 28 | 18 | M | Upper tibia* |
| 29 | 28 | F | Upper ulna |

* Lung metastases.

shows the sites. All these tumours arose in long bones, 4 in the tibia, and 4 around the knee. The figures reveal a surprising number of fibroblastic bone tumours in Uganda African children. This histological pattern was fairly constant in all the cases—fibre-forming spindle-cell tumours, showing cellular pleomorphism, frequent mitoses, and tumour giant cells (Fig. 7).

*Chondroma and chondrosarcoma.*—Seven tumours producing chondroid tissue, but without areas of undifferentiated mesenchymal tumour or of osteo- or fibrogenesis were seen (Table IV) ; as often, it was difficult to assess the degree of

TABLE IV.—*Chondromatous Tumours in Uganda Africans*

| Case Number | Age | Sex | Site | Duration | Histology |
|---|---|---|---|---|---|
| 30 | 16 | M | Buttock | 1 mo. | Chondrosarcoma |
| 31 | 16 | M | Ilium | 8 yr. | Calcifying. Doubtful malignancy. |
| 32 | 19 | M | Upper femur | 6 mo. | As case 31 |
| 33 | 23 | M | Skull | 1 yr. | Low grade malignancy |
| 34 | Adult | F | " Foot " | ? | Highly calcified. Doubtful malignancy |
| 35 | Adult | M | Lower femur | ? | Histologically benign |
| 36 | Adult | M | Thumb | 3 yr. | ,, ,, |

malignancy of several of these tumours. At least 3 had been present for a year when the patients were first seen, and three are graded as benign histologically. Only one was certainly a phalangeal chondroma. Three tumours were extensively calcified (Fig. 8).

*Giant-cell tumour.*—Only 3 acceptable cases were recorded (Table 5 and Fig. 9). These corresponded in age incidence, site and histological pattern with the giant-cell tumours (osteoclastomas) seen elsewhere. One case, in a girl of 11, was

TABLE V.—*Giant-cell Tumours in Uganda Africans*

| Case number | Age | Sex | Site |
|---|---|---|---|
| 37 | 20 | M | Lower end femur |
| 38 | 32 | M | ,, ,, ,, |
| 39 | 40 | M | ,, ,, radius |

originally diagnosed as a giant-cell tumour, but is now believed to be an aneurysmal bone cyst. This was a cystic lesion " expanding " the calcaneum, and the cyst wall showed a reactive type of fibrous tissue, with many osteoclast-like cells projecting into the cyst cavity (Fig. 10). This lesion should not, perhaps, be classed as a neoplasm, but is included here because of its resemblance to, and confusion with, giant-cell tumour.

*Plasmacytoma and myelomatosis.*—Nine cases of plasmacytic tumours in bone were recorded. Unfortunately, evidence for myelomatosis had not been sought in every case, although in at least two cases the diagnosis of multiple myeloma seems almost certain (cases 48 and 49). Details of the cases are given in Table VI. It

TABLE VI.—*Plasma-cell Tumours of Bone in Uganda Africans*

| Case number | Age | Sex | Site | Evidence of myelomatosis |
|---|---|---|---|---|
| 41 | 20 | M | Femur | No evidence |
| 42 | 25 | M | Foot and tibia | More than one bone involved |
| 43 | 25 | M | Sacrum | No evidence |
| 44 | 30 | F | Tibia | ,, , |
| 45 | 38 | M | Humerus | ,, ,, |
| 46 | 40 | M | Femur | ,, ,, |
| 47 | 42 | M | Spine | Extra-dural tumour, T.12-L.4. Paraplegia |
| 48 | 50 | M | Sternum | Osteolytic areas in skull. Serum globulin 5·4 g./100 ml. |
| 49 | 56 | M | Rib | Osteolytic areas in ribs and ilia. |

will be noted that the plasmacytomas occur at a higher mean age than the other groups, and that long bones were reported involved in only 5 out of 9 cases. There was no evidence of amyloidosis in any of the cases.

*Other tumours.*—These comprise 2 osteochondromatous masses (probably exostoses), 2 probable lymphomas (both involving the femur), and 2 unclassifiable anaplastic tumours. Among primary bone tumours unrepresented in this series are osteoid osteoma, non-ossifying fibroma, and Ewing's tumour. Cases of Burkitt lymphoma and of tumours metastatic in bone were excluded. So also were numerous cases of carcinoma arising in tropical ulcer, of Kaposi Sarcoma, or of malignant melanoma, with invasion of underlying bone.

*Tribal distribution.*—Twenty seven of the 56 cases were filed in the Kampala Cancer Registry, and in these the patient's tribe is recorded. Only seven were members of the Ganda tribe. In the 29 Uganda Government Laboratory cases,

---

### EXPLANATION OF PLATES

(All photomicrographs are of H. & E.-stained sections)

FIG. 1.—Fibrosarcomatous pattern in an osteogenic sarcoma in a girl of 12 years. × 85.
FIG. 2.—Tumour osteoid showing calcification in an osteogenic sarcoma of the skull. × 205.
FIG. 3.—Areas of tumour bone, and several osteoclasts, in an otherwise undifferentiated sarcoma. × 85.
FIG. 4.—Cartilaginous differentiation in an osteogenic sarcoma. × 205.
FIG. 5.—Tumour osteoid in an osteogenic sarcoma (same case as Fig. 5). × 205.
FIG. 6.—Large areas of tumour bone in a sclerosing osteogenic sarcoma. × 85.
FIG. 7.—Fibrosarcoma of bone. × 85.
FIG. 8.—Low-grade chondrosarcoma showing areas of calcification. × 85.
FIG. 9.—Giant-cell tumour of bone. × 205.
FIG. 10.—Aneurysmal bone cyst. Fibrous cyst wall with many osteoclast-like cells lining its inner margin. × 85.

1

2

3

4

5

Dodge.

6

7

8

9

10

Dodge.

the tribe is usually not given, but the location of the referring hospital is known. Eight of these 29 cases were referred from hospitals in Buganda Province (and this includes Mulago Hospital, the teaching hospital of Makerere College Medical School, to which cases are referred from the whole of Uganda). At most, therefore, 15 out of 56 (27 per cent) of the primary bone tumours occurred among Ganda tribespeople. Full figures are given in Table VII. Numerous other tribes and areas of Uganda are represented in the series, but none in disproportionate numbers.

TABLE VII.—*Tribal and Geographical Distribution of* 56 *Cases of Skeletal Tumours in Uganda Africans*

| | Tribal origin (Cancer Registry cases) | | Hospital origin (Govt. Laboratory cases) | |
|---|---|---|---|---|
| | Ganda | Other | Buganda (including Mulago) | Other |
| Osteogenic sarcoma | 0 | 10 | 3 | 9 |
| Fibrosarcoma | 1 | 2 | 0 | 4 |
| Chondroma and chondrosarcoma | 2 | 1 | 1 | 3 |
| Giant-cell tumour | 2 | 0 | 0 | 1 |
| Plasmacytoma | 0 | 7 | 0 | 2 |
| Other tumours | 2 | 0 | 4 | 2 |
| | 7 | 20 | 8 | 21 |

*Clinical cases.*—A diagnosis of primary bone tumour was made in sixteen cases where histological confirmation was lacking. Radiological studies were made in all 16 cases, and the diagnosis of bone tumour is well substantiated in 15 cases (in one, tuberculosis was not excluded). The femur was the bone involved in 10 cases. The radiological diagnosis was osteogenic sarcoma in 9 cases, fibrosarcoma, chondro-sarcoma and giant-cell tumour in one case each, unspecified in four. The youngest patient was 7 years old, the oldest 60 years.

*Tumours in Uganda Asians.*—Only two primary skeletal tumours among Asians were recorded. One was a giant-cell tumour of tibia, and one a liposarcoma of femur. None of the commoner types of bone tumour was represented.

### DISCUSSION

*Frequency of bone tumours in Africans.*—In an earlier report from Uganda (Davies and Davies, 1960), bone tumours were found to form 5·2 per cent of all malignant tumours (120 cases). But of these, no less than 86 (3·6 per cent) were jaw tumours (mostly adamantinomas and multicentric sarcoma—now designated as Burkitt lymphoma). The 34 tumours of other bones (1·6 per cent of all malignant tumours) included 15 osteosarcomas and 11 cases of myeloma.

In Kenya a survey of 2747 cases of neoplastic disease (Linsell and Martyn, 1962) revealed 24 cases of bone sarcoma, 14 osteoclastomas and 6 myelomas (44 cases, 1·5 per cent of all malignant disease). This excludes 12 cases of adamantinoma.

In their survey of malignant disease in Africans in Transvaal, South Africa, Higginson and Oettlé (1960) found that bone tumours were less frequent than among the U.S. population.

In the period covered by the present survey, the Kampala Cancer Registry recorded 3172 cases, and the 27 verified bone tumours represent 0·85 per cent of this total. The main types of malignant bone tumour are represented, with the exception of Ewing's tumour and Paget's sarcoma. Benign tumours are poorly represented ; in Africa, it is only the incapacitating lesions that are likely to bring the patient to hospital. Only 13 patients were willing, or in a fit condition, to undergo excisional surgery. Permission to amputate a limb is often refused, and no radiotherapy is available in East Africa. (Some recent cases of osteogenic sarcoma have received intra-arterial chemotherapy). The histological material was therefore limited, and perhaps unrepresentative in some cases.

*Bone tumours in children.*—Eight bone tumours were seen in children under 15 years, and five of these are classed as fibrosarcomas. It is debatable whether these are true fibrosarcomas of bone or fibroblastic osteogenic sarcomas in which limited biopsies do not reveal areas of osteoid formation. Fibrosarcoma of bone was not a common tumour in Dahlin's American series (Dahlin, 1957). He recorded no cases in the first decade and the largest number of his cases occur in the fourth decade ; the fibroblastic type of osteogenic sarcoma also manifested itself at a higher mean age than other types. It seems that, however one classifies them, fibroblastic malignant tumours of bone appear to be more prominent in Uganda African children than in those of Western countries. In Dahlin's series, fibrosarcomas and fibroblastic osteogenic sarcoma together make up 13 per cent of the malignant bone tumours occurring in the first 2 decades. In Uganda, they account for 7 out of 17 (40 per cent) of malignant bone tumours in these age-groups. The sex ratio of 4 : 3 in these Uganda fibrosarcomas contrasts with that of 16 : 6 in the osteogenic sarcomas. It is tempting to relate this early onset of bone sarcoma in Uganda children to the known differences between the bone growth rates of African and Western children. In the first few months of life, African babies gain in weight and length more rapidly than European children, and show more rapid skeletal maturation (Trowell, 1960). This precocious growth persists, to a lessening degree, in children of 1–3 years, but gives way to a relative retardation in the 5–10 year age-group. The retardation may well be the result of a protein-poor diet. In kwashiorkor there is considerable retardation of bone growth, which is well-marked at the lower end of the femur (Jones and Dean, 1959). Neither rickets nor scurvy is often seen in African children in Uganda.

*Osteogenic sarcoma.*—The 22 cases seem to resemble, in age and site distribution and in histological pattern, other series of this tumour. It is interesting that Paget's disease of bone has never, to the author's knowledge, been seen in a Uganda African, in spite of being specifically sought in many hundreds of necropsies. However, at least 3 of the 22 osteogenic sarcomas occurred in patients aged 40 or over. *Chondroma* and *chondrosarcoma,* and *giant-cell tumour of bone* appear to manifest themselves much as in Europeans.

*Plasmacytoma and myelomatosis.*—Isolated soft-tissue plasmacytomas are by no means rare tumours in Uganda Africans. At least 2 of the plasma-cell tumours in this series were accompanied by evidence of multiple myelomatosis. Plasma cells figure prominently in the histopathology of the African, and they are associated with a high level of plasma gamma-globulins, which rise above normal European levels as early as 6 months after birth, and may reach levels of 2 g. or more per 100 ml. (Trowell, 1960).

*Tribal distribution.*—It is not clear why Ganda tribespeople are so under-represented in this series of bone tumours. Kampala, Uganda's capital and the site of the Medical School, lies in the Province of Buganda. The Ganda, who make up

16 per cent of the population of Uganda as a whole, constitute some 60 per cent of the population of the Kampala area. They form the most economically and educationally advanced segment of Uganda's peoples. These facts are reflected in the over-representation of this tribe in most disease surveys based on Kampala hospital admissions. Over 60 per cent of all tumours in the Kampala Cancer Registry are from Ganda patients, and the proportion remains similar when various tumour sites are considered separately—i.e. 52 per cent of penile cancers (Dodge and Linsell, 1963), 60 per cent of prostate cancers (Dodge, 1963), 71 per cent of cervical cancers (Dodge, *et al.*, (1963), 50 per cent of salivary tumours (Davies, *et al.*, 1964). A similar dearth of cases from Buganda Province is apparent in the Government Laboratory series, where the patients are grouped by location of referring hospital, but not by tribe. The shortage of Ganda patients applies to tumours as different as plasmacytoma and osteogenic sarcoma.

In the case of carcinoma of penis and prostate, it was possible to work out age-specific incidence rates for Kyadondo county (the district surrounding and including Kampala), based on a recent census (Davies, *et al.*, 1962). In the present series, only six cases were reported from Kyadondo, and 3 of these were in immigrants. No meaningful incidence rates can therefore be calculated for bone tumours.

SUMMARY

Bone tumours (other than tumours of the jaw) account for about 1 per cent of malignant tumours in Uganda Africans. This paper analyses 56 histologically confirmed cases. The commonest tumour encountered is osteogenic sarcoma, followed by plasmacytoma.

A striking feature is the occurrence of fibroblastic sarcomas in the long bones of children under 15 years.

Analysis of the tribal distribution reveals an unexplained dearth of skeletal tumours among the Ganda tribe.

I would like to express my gratitude to the late Professor D. H. Collins for his helpful advice in preparing this paper.

### REFERENCES

ACKERMAN, L. V. AND SPJUT, H. J.—(1962) ' Tumors of bone and cartilage : Atlas of Tumor Pathology '. Washington, (Armed Forces Institute of Pathology).
BURKITT, D.—(1958) *Brit. J. Surg.*, **46**, 218.—(1963) *Int. Rev. exp. Path.*, **2**, 69.
DAHLIN, D. C.—(1957) ' Bone Tumors '. Springfield. (Charles C. Thomas.)
DAVIES, A. G. M. AND DAVIES, J. N. P.—(1960) *Acta Un. int. Cancr.*, **16**, 1320.
DAVIES, J. N. P., DODGE, O. G. AND BURKITT, D.—(1964) *Cancer*, **17**, 1310.
*Idem*, WILSON, B. A. AND KNOWELDEN, J.—(1958) *Brit. med. J.*, ii, 439.—(1962) *Lancet*, ii, 328.
DODGE, O. G.—(1963) *Cancer*, **16**, 1264.
*Idem* AND LINSELL, C. A.—(1963) *Ibid.*, **16**, 1255.
*Iidem* AND DAVIES, J. N. P.—(1963) *E. Afr. med. J.*, **40**, 440.
HIGGINSON, J. AND OETTLÉ, A. G.—(1960) *J. nat. Cancer Inst.*, **24**, 589.
JAFFE, H. L.—(1958) ' Tumors and tumorous conditions of the bones and joints '. London (H. Kimpton).
JONES, P. R. M. AND DEAN, R. F. A.—(1959) *J. Pediatrics*, **54**, 176.
LINSELL, C. A. AND MARTYN, R.—(1962) *E. Afr. med. J.*, **39**, 642.
TROWELL, H. C.—(1960) ' Non-infective disease in Africa '. London (E. Arnold.)